# A Timely Intervention: Endoscopic Retrieval of a Swallowed Magnetized Activity Watch

**DOI:** 10.1155/2016/2190726

**Published:** 2016-01-19

**Authors:** Jason S. Radowsky, Joseph S. Lee, Andrew T. Schlussel

**Affiliations:** Department of General Surgery, Brian Allgood Army Community Hospital, Yongsan 96205, Republic of Korea

## Abstract

The accidental ingestion of a foreign object often presents a difficult scenario for the clinician. This includes not only the decision to retrieve the material but also the appropriate technique to use. We present the case of a young asymptomatic girl who swallowed a magnetic activity watch, which was then successfully retrieved with an endoscopic snare. To our knowledge, this is the first documented case of salvaging an operational watch from the stomach using an endoscopic technique.

## 1. Introduction

Foreign body ingestion is a challenging medical dilemma for both surgeons and gastroenterologists, with the greatest incidence seen in the psychiatric, elderly, and pediatric populations. The mechanism of ingestion varies based on the cohort studied. The psychiatric population may have an altered decision-making capability or will swallow objects for a secondary gain [1]. The elderly often have altered mental status, decreased oropharyngeal sensation and control, and ill-fitting dental appliances. The vast majority of cases, > 80%, occur in children. However, only 10–20% require endoscopic removal, with < 1% mandating an operation to remove the foreign body [2, 3]. Younger children demonstrate the greatest risk as their senses are developing and begin to explore their own environment, frequently placing objects in their mouth [4]. The object ingested, anatomical location, and age of the patient are important factors when making the clinical decision to pursue extraction through an invasive technique or to manage the patient expectantly in hopes of transit through the gastrointestinal tract [5]. An endoscopic or operative approach to extract dentures, batteries, or magnets is strongly recommended, as these objects portend a greater risk of intestinal laceration, perforation, obstruction, or luminal necrosis [4, 6, 7].

As one may expect, there is a paucity of data in the literature regarding the extraction of an ingested watch or object with similar dimensions. One case report has discussed the extraction of a watch through an open gastrostomy, three cases have utilized an endoscopic approach for retrieval of an impacted watch in the proximal esophagus, and one report discussed successive esophageal balloon dilation, to dislodge the timepiece, pushing it distally into the stomach to allow for passage [8–11]. To our knowledge this is the first documented case of a watch extraction from the stomach using a flexible endoscope.

## 2. Case Report

**Figure 1:**
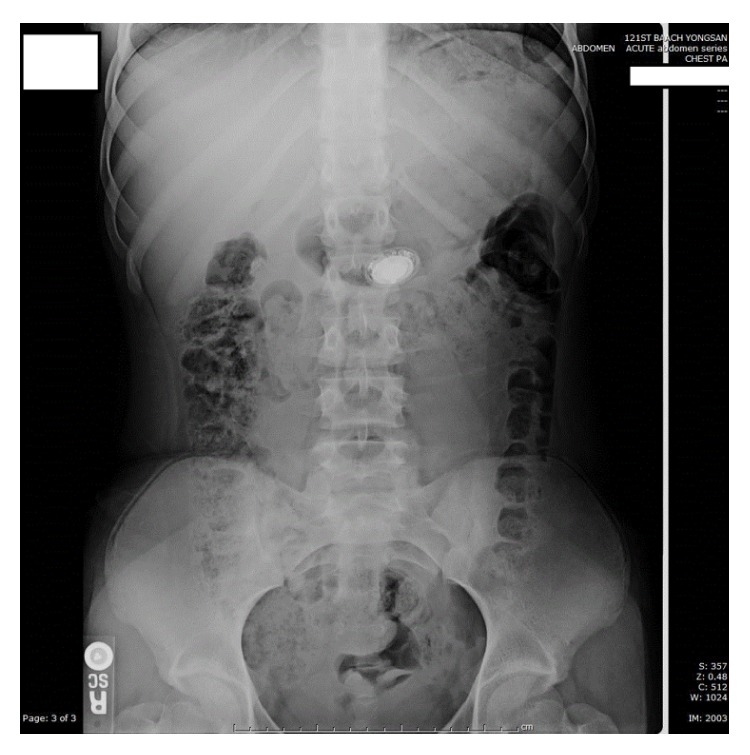
Supine abdominal X-ray showing watch in the stomach.

**Figure 2:**
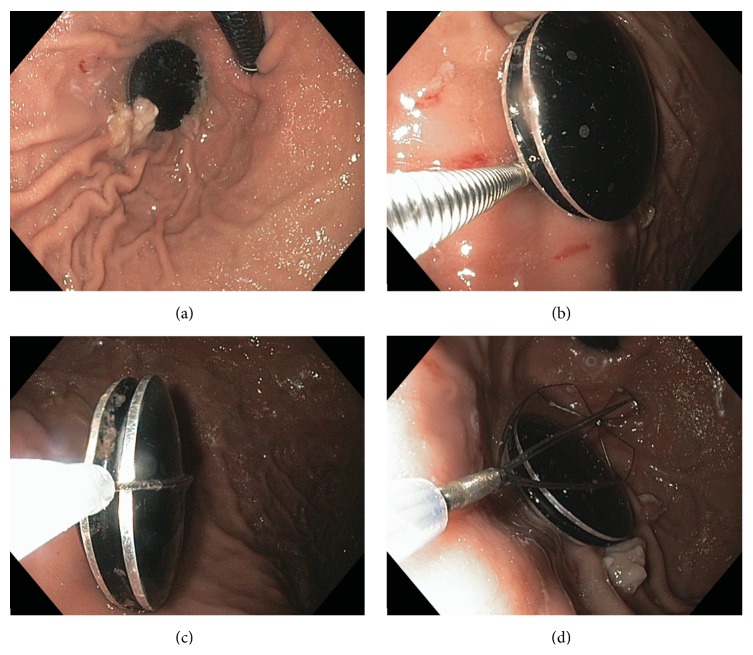
Watch shown in gastric fundus on retroflexion (a) and failed attempts at retrieval using biopsy forceps (b), transverse capture with a snare (c), and wire basket (d).

**Figure 3:**
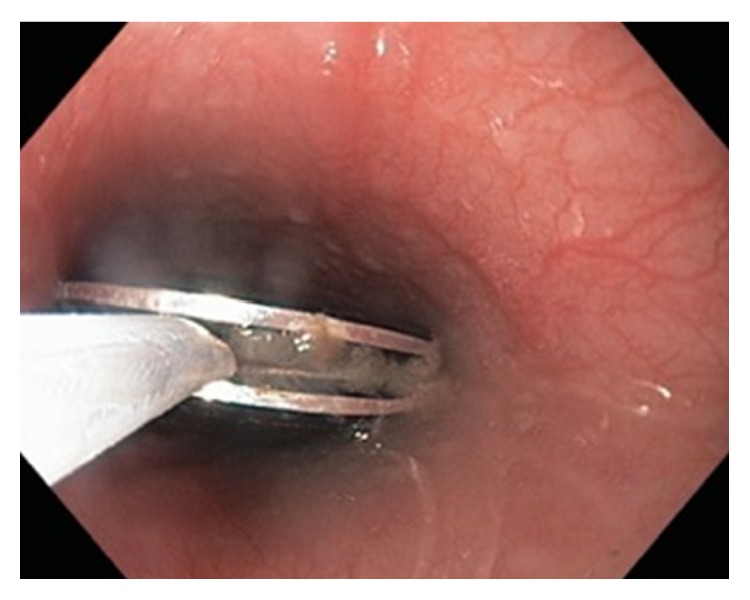
Successful end-on lassoing in the groove of the watch with the snare.

This is a case of a healthy, well-developed, 13-year-old girl who was reported to have accidentally swallowed a Shine Misfit fitness tracking watch (Misfit Wearables, Burlingame, CA, USA). While swimming, she removed the magnetized functional portion of the watch from its band and placed this in her mouth at which time it was swallowed by mistake. At the time of presentation, the object was identified in the stomach on an emergency department X-ray at our community-sized hospital. The patient was initially managed conservatively and was informed to return the following day for serial imaging (Figure 1). After approximately 30 hours, the watch was still retained in the stomach. Due to the failure of distal progression through the pylorus and concern for obstruction at the ileocecal valve, the decision was made to proceed with an endoscopic intervention in the operating room. The watch was magnetized, measuring 28.5 × 8.0 × 28.5 mm (width × depth × height), which precluded grasping the device [12]. The size and smooth oval shape of the watch made capturing it with a wire basket or endoscopic snare difficult (Figure 2). Finally, with no additional interventional equipment available, a snare was used to manipulate the device into the esophagus, where it was then secured utilizing an end-on lassoing technique around the groove of the watch. Once the device was fully encircled, it could then be safely removed through the oropharynx (Figure 3). Following this 72 min procedure, the patient recovered well and was discharged home the following day with no apparent sequela from the event. The watch retained normal function despite the low pH of the stomach and manipulation upon retrieval. When synchronized to her mobile device, the Shine Misfit watch accurately recorded all advertised data points to include steps taken, calories burned, sleep cycles, and maintained accurate time. The patient's father provided consent for the use of the images and information in this case report.

## 3. Discussion

The accidental ingestion of a foreign object is far more common in pediatric, elderly, psychiatric, and incarcerated patients when compared to the adolescent or healthy adult population. Once in the stomach, 80% of these objects will eventually pass out of the body; therefore, intervention remains uncommon as long as the patient is asymptomatic [13]. The decision to proceed with endoscopic retrieval, in this case, was prompted by a failure in the progression of the watch for greater than 24 hours. In addition, there was an uncertainty to the maintenance of the watch's structural integrity when exposed to a low pH for an extended period of time. The device is advertised as being waterproof to 50 meters; however, this did not allay the surgeon or patients' concern for battery exposure and the risk of a caustic perforation [12]. Several studies have shown that sharp foreign bodies, severe symptoms, long duration from ingestion to endoscopy, and existence of mucosal injury are significant risk factors predictive of complications related to removal of foreign bodies [14, 15]. We believe that early detection and a timely endoscopic removal were two factors that contributed to successful retrieval for this case.

As this retrieval occurred at a small community hospital, a limited variety of endoscopic tools were available. While there are no published guidelines for which endoscopic snare or grasper to utilize during foreign body recovery, the American Society for Gastrointestinal Endoscopy (ASGE) provides recommendations regarding the dimensions, configurations, and indications for many commercially available devices [16]. In addition, the ASGE has published intervention timing guidelines based on patient symptoms, object type and size, and anatomic location. Although a magnetized digital watch is not included in these published categories, disk batteries and coins larger than 2 cm are recommended to be extracted 1-2 days following ingestion [17].

In conclusion, the retrieval of a magnetized watch from the stomach with a flexible endoscope is a safe and efficacious procedure. It is critical that the physician must consider patient size, as well as the device size and design, to formulate an appropriate management plan. The patient should understand the endoscopic and operative risks and should be counseled on the need for a timely intervention when observation has failed.

## Copyright Protection

Our team is comprised of military service members and employees of the US Government. This work was prepared as part of our official duties. Title 17 U.S.C. 105 provides that “Copyright protection under this title is not available for any work of the United States Government.” Title 17 U.S.C. 101 defines a US Government work as a work prepared by a military service member or employee of the US Government as part of that person's official duties.
